# Decoding dynamic emotional valence in GenAI interactions: insights from covariate-dependent Markov chains

**DOI:** 10.3389/fpsyg.2026.1783364

**Published:** 2026-05-11

**Authors:** Yiming Taclis Luo, Ting Liu, Patrick Pang, Ho Yin Kan

**Affiliations:** Faculty of Applied Sciences, Macao Polytechnic University, Macao, China

**Keywords:** AI response quality, emotional stability, human–GenAI interaction, Markov chain, process modeling

## Abstract

**Introduction:**

User affective valence states evolve dynamically during human–GenAI interaction, yet existing research provides limited insight into how the quality of AI outputs is associated with these moment-to-moment emotional valence transitions.

**Objective:**

This study aims to fill this gap by proposing a covariate-dependent Markov chain model to examine how the quality of AI responses is associated with transitions in user emotional valence.

**Methods:**

We conducted an experiment on AI-assisted academic writing for university students and analyzed 886 interaction sequences.

**Results:**

The results show a significant polarization effect of AI response quality on user emotional valence state transitions: high-quality responses stabilize and reinforce positive emotional valence, while low-quality responses tend to trigger emotional deterioration. Furthermore, group differences based on emotional stability are also analyzed.

**Discussion:**

This research provides a new perspective for understanding emotional dynamics in human–computer dialogues and offers practical evidence for building emotionally adaptive GenAI systems.

## 1 Introduction

Emotion is a person’s immediate reaction to current events, and it is one of the most important factors influencing human–computer interaction (Lottridge et al., 2011). Most of the current research on human–computer interaction emotions uses classic emotion theories as the framework for emotion classification, such as the six basic emotion theories (Ortony, 2022) and Dimensional Models of Emotion (Reisenzein, 1994). These classic emotion classification theories have been deployed in a wide range of research that requires the support of affective computing. Although these emotion classification models occupy a major position, emotion recognition based on situational dependence and construction has also received increasing attention in recent years.

Cognitive evaluation theory believes that emotions are not directly caused by the situation itself but are caused by the individual’s subjective interpretation, evaluation, and judgment of the situation (Rummel and Feinbero, 1988). It emphasizes the complexity of emotional categories in individual states. For example, prior research Cowen and Keltner, empirically demonstrates that specific emotions are unique in certain contexts, such as watching videos or listening to music (Cowen and Keltner, 2020). Similarly, research in the science of learning has identified nine specific learning emotions that are unique to classroom and exam situations (Pekrun and Linnenbrink-Garcia, 2012). The cognitive-evaluative perspective, which emphasizes subjective interpretation, naturally leads to the necessity of studying emotional dynamics, as an interaction is a continuous chain of changing situations and subsequent evaluations. With the increasing complexity of human–computer collaboration, traditional static snapshots of emotions are no longer sufficient to capture the full picture of the user experience. Research is shifting toward continuous tracking and modeling of emotional dynamics, particularly in scenarios that emphasize ongoing interaction.

This shift reflects a human–centered philosophy to create a more supportive and responsive interactive environment (Ma et al., 2024). The field of education is a key context for studying such emotional dynamics. The learning process itself is an activity with high emotional investment, goal orientation, and cognitive challenges. Emotions have always been a core factor affecting learners’ learning outcomes in human–computer interaction environments. Positive emotions can significantly enhance learners’ motivation, engagement, and creativity, thereby improving their academic performance (Li et al., 2020; Oriol et al., 2016). In recent years, educational research has increasingly emphasized the importance of emotional factors and is committed to improving the quality of interaction between students and systems through emotion recognition and intervention to meet students’ personalized learning needs (Salloum et al., 2025; Vistorte et al., 2024). For example, Dake and Gyimah effectively identified and understood learners’ emotions and attitudes by analyzing a large amount of text feedback generated by students in online learning systems, thereby providing support for improving teaching quality (Dake and Gyimah, 2023).

With the rise of Generative Artificial Intelligence (GenAI), learning is transforming into a personalized, interactive process centered on human–computer dialogue (Chou et al., 2022). Within human–GenAI interactions, the primary stimulus that triggers learners’ cognitive evaluations is the quality of the AI’s output. The quality of the AI’s output, including its relevance and reliability, acts as the immediate, context-specific stimulus that triggers the learner’s emotional valence change and subsequent cognitive reappraisal (Hu and Shao, 2025). Unlike traditional systems, human–computer dialogue involves a real-time, continuous feedback loop (Luo et al., 2026; Luo et al., 2025). As cognitive appraisal theory suggests, the immediate feedback loop in dialogue is closely associated with affective polarity transitions, reflecting the user’s instantaneous evaluation of whether the GenAI’s response facilitates or hinders their goals (Roseman and Smith, 2001).

Therefore, emotional dynamics in human–GenAI dialogue cannot be understood without examining the quality of AI responses as a key correlation of affective valence transitions.

However, despite the central role of GenAI response quality in shaping learners’ moment-to-moment appraisals, existing research rarely examines how variations in AI output quality are associated with affective valence transitions during real-time interaction. One gap is that many human–GenAI interaction studies use self-assessment scales for evaluation, lacking more fine-grained measurement and understanding of changes in these emotional and cognitive processes (Wu and Liu, 2025; Wu et al., 2025). In addition, mainstream research tends to focus on the impact of different presentation formats of GenAI on users’ ultimate outcomes, while lacking a detailed and in-depth exploration of the dynamic interaction process between the learner and the GenAI. For instance, studies often use scales to explore the effects of various types of GenAI feedback on learners (Guo and Wang, 2025; Zou et al., 2025), rather than concentrating on the feedback details and behaviors during the real-time process of interaction between the GenAI and the student. The ability to accurately measure dynamic emotions is crucial to supporting personalized learning and strategy implementation in GenAI-assisted environments. This will not only help us build a more adaptive and empathetic education system, optimize the learning experience by identifying and responding to students’ emotional changes, but also provide robust data-driven insights for the improvement of teaching strategies and content (Ma et al., 2025).

To address this gap, the present study investigates the associations between variations in GenAI output quality and learners’ emotional changes during academic writing tasks, focusing on emotional valence, which represents the most fundamental dimension of affective appraisal in human–AI collaboration (Shuman et al., 2013).” We map emotional valence into three dimensions: positive, negative, and neutral, and conceptualize it as the emotional state in this paper. Conducted with university students in a GenAI-assisted learning environment, the study examines emotional dynamics at the turn-by-turn level of human–AI dialogue, capturing how each AI response influences the learner’s subsequent emotional valence.

We develop a covariate-dependent Markov chain model that treats emotional valence transitions as temporally dependent events influenced by the quality of preceding AI responses. Emotion transitions serve as the dependent behavioral indicators of learners’ evolving affective states, while AI response quality is incorporated as a key interaction covariate expected to modulate the probability of specific emotional shifts. We used multinomial logistic regression to fit the model, statistically quantifying the dynamic regulatory effect of AI response quality on the probability of specific emotional valence transitions.

To establish a robust empirical foundation for this model, we conducted a GenAI-assisted learning experiment with university students focused on academic writing tasks. We employed a mixed-method design that integrates multidimensional data collection (conversation logs, screen recordings, and retrospective interviews) and collected 881 sets of continuous interaction records (including the previous emotional state, the quality of the AI response, and the next emotional state). Our research discretizes users’ emotional valence into three core states: Positive, Negative, and Neutral, which are calculated by combining users’ prompts and interview text on a fine-tuned large language model. AI response quality is defined using a multi-dimensional construct covering five key dimensions (Relevance, Reliability, Transparency, Fairness, and Criticality) and assessed and classified via human expert consensus. Finally, to eliminate the impact of heterogeneity (inherent differences among individuals), we assessed users’ emotional stability and used a data-driven grouping method (SVD and K-means clustering) to divide participants for targeted analysis.

By integrating these analytical methods, this paper provides a dynamic perspective to comprehensively understand how human–computer interaction factors affect learners’ emotional changes. In summary, we raise the following questions:

RQ1: What are the patterns of emotional state transitions during human–computer interaction?

RQ2: How does AI’s response in human–computer interaction influence the emotional state transitions with different emotional stability groups?

RQ3: What implications do the process findings have for the design of adaptive emotional support and teaching interventions in a human–computer interaction environment?

By examining the fine-grained emotional dynamics within this continuous-interaction scenario, this study provides a solid theoretical and practical foundation for designing more humane and adaptive human–computer interaction systems and emotional support strategies.

## 2 Literature review

### 2.1 The role of emotion in human–AI interaction

A range of emerging research indicates that emotional factors are crucial for establishing effective, natural, and satisfying collaborative relationships between humans and AI. It has been widely verified that emotion is a key factor affecting the quality of interaction and behavioral intention between humans and AI (Kolomaznik et al., 2024) emphasize the importance of “social–emotional attributes” such as trust, empathy, and rapport in enhancing human–AI collaboration, arguing that these factors are the cornerstones of effective interactions. Yao et al. found that considering emotion can more comprehensively consider the various factors of an interaction, rather than simply rational trust assessments (Yao et al., 2024). A study further demonstrated that understanding emotions is key to effective collaboration in “hybrid teams” involving multiple humans and AI systems (Ferrada and Camarinha-Matos, 2024). This suggests that the influence of emotions is not limited to the interaction between a single person and AI but extends to complex ecosystems involving multiple parties collaborating.

In addition, the feedback loop of emotions in the interaction between humans and AI will change human cognition and judgment. This demonstrates that AI’s responses also play an important role in understanding human emotion. The research of Glickman and Sharot revealed how this feedback loop affects human perception, emotion and social judgment, and may even lead to judgment bias (Glickman and Sharot, 2025). They also called for considering not only how AI responds to human emotions when designing AI systems, but also how AI’s output in turn shapes human cognitive processes. The importance of this feedback loop is also reflected in more complex collaborative environments. Dang et al. examined how cognitive and social–emotional interactions work together to change their cognition and promote learning outcomes in their study of human–AI collaborative learning in a mixed reality environment (Dang et al., 2025) This further shows that the role of AI in collaboration is no longer just to provide information, but also to interact with humans’ emotional states to improve collaborative efficiency and results. Therefore, this provides a theoretical basis for incorporating AI response quality as a covariate into the human–computer interaction framework of this study.

### 2.2 Learning sentiment analysis

Sentiment analysis, as an effective data mining technique, is increasingly being used in the field of learning analytics and has become an active research topic. This technology can automatically identify, extract, and quantify subjective sentiment within text, providing insights for educators and institutions to improve teaching quality, optimize course design, and enhance the learner experience. Numerous review studies have highlighted the enormous potential of sentiment analysis in education (Altrabsheh et al., 2013). A survey also indicates that educational institutions have invested extensively in developing sentiment analysis tools to process and analyze student feedback data (Shaik et al., 2023). By analyzing this data, educational institutions can identify student satisfaction, emotions, and opinions, enabling them to make more informed decisions (Pooja and Bhalla, 2022).

Research methods for educational sentiment analysis are constantly changing and evolving. Some studies rely mainly on artificial intelligence methods, such as using machine learning to conduct sentiment analysis on distance education course materials to assess student perceptions (Osmanoğlu et al., 2020). In recent years, with the development of deep learning technology, researchers have begun to utilize deep neural network models to analyze text data to improve analysis accuracy (Kastrati et al., 2021; Shuqin and Raga, 2024).

Considering that traditional learning sentiment analysis typically provides static snapshots of emotions and lacks an in-depth understanding of the dynamic changes in emotions, a series of studies have begun to explore its time series analysis. By analyzing and tracking over time, researchers can more comprehensively capture and understand students’ emotional evolution, patterns, and trends during the learning process. The sentiment detection methods under time series are divided into invasive and non-invasive. Invasive methods typically require users to wear specific devices to directly measure physiological signals, which are considered objective indicators of emotion. This method can provide a certain degree of accurate data but may cause discomfort to users or affect their natural behavior. For example, Mikuckas et al. used the time series of electrocardiogram signal intervals to identify students’ emotions (Mikuckas et al., 2014). An interval time series of electrocardiogram signals to identify students’ emotions. Non-intrusive methods infer emotions by observing the user’s external behavior or digital traces, including using images, text, and device interaction information, without the user having to wear any special equipment. This method is generally more natural and easier to deploy in real-world environments. For example (Shou et al., 2023) proposed a sentiment analysis method based on classroom time series images, which comprehensively portrays students’ emotional states by analyzing a series of continuous classroom images. This method can more comprehensively reflect the true emotional dynamics in the classroom.

### 2.3 Human**–**computer interaction process modeling

Mental models are a crucial theoretical foundation for understanding human**–**computer interaction. They refer to users’ internal representations or beliefs about a system’s internal workings and operational logic. Great human**–**computer interaction design aims to help users build a stable, complete, and easily understood mental model, enabling them to effectively predict system outputs, solve problems, and complete tasks. Therefore, modeling the interaction process is largely an exploration of how users construct, adjust, and apply mental models.

A common method is to use post-interaction surveys to assess the user’s overall satisfaction and final emotional state to infer the user’s mental model status. For example, Du and Reynolds et al. used a long-term controlled experiment to explore how the interaction dynamics mediated by AI induce changes in learning motivation over time (Du and Reynolds, 2025). Jasin et al. collected qualitative interview and self-report data from students to document their emotions, experiences and perspectives during the interaction (Jasin et al., 2023). However, these methods have some limitations. For example, they can only capture users’ subjective feelings or final states, and the accuracy may be affected by memory bias.

To address these limitations, interactive behavior and operation log analysis have become a mainstream approach. For instance, lag sequence analysis can be applied to understand interaction sequences in visualizations (Pohl et al., 2016), and machine learning and sequence analysis can be used to analyze the dynamic changes in user emotions and cognition (Ma et al., 2025). These methods record every user’s action, pattern, and time in the system in real time, converting the formation and evolution of mental models into quantitative metrics to facilitate subsequent modeling and analysis.

However, these existing methods are often based on simple sequence statistics, making it difficult to accurately capture the dynamic changes in the user’s mental model state under the influence of different factors (including categorical variables and covariates). Building on these dynamic capture methods, this paper further proposes the covariate-dependent Markov chain model. Its core advantage is that by directly incorporating covariates into the model, it can more accurately model and describe the dynamic evolution of human**–**computer interaction and the driving factors behind it, addressing potential shortcomings of traditional methods in describing dynamics and influencing factors.

## 3 Methodology

This section introduces the proposed experimental and covariate-dependent Markov chain model, as well as some analyses to satisfy the model’s prerequisites.

### 3.1 Experimental design

Conducting controlled experiments in simulated human**–**computer dialogue environments to observe the dynamic impact of AI response quality on users’ emotional states is crucial. Experimental design should ensure that tasks closely resemble real-world interaction scenarios, allowing users to generate natural emotional responses during interaction with the AI. This requires task design to be sufficiently robust, open, and complex, requiring different users to complete multiple rounds of dialogue in a non-specific AI environment to achieve their goals. This allows for the observation of dynamic changes in emotional states over the course of continuous interaction.

This study designed an academic writing task in an experimental setting where participants were recruited to complete the task individually in a laboratory. We designed a conversational task, and participants were randomly assigned a conversational AI system to complete a series of practical tasks (see Appendix A for specific scenarios and requirements). After the experiment, we collected multi-dimensional interaction data and conducted semi-structured interviews with participants. At the end of the experiment, all participants received a small token of appreciation. This study received ethical approval from the Ethics Committee of Macao Polytechnic University (Ethics Approval No.: HEA006-FCA-2025, approved on 6 June 2025).

### 3.2 Pre-test questionnaire and group classification method

Emotional stability, a core dimension of the Five-Factor Model of personality (McCrae and Costa, 2008), refers to an individual’s capacity to remain psychologically composed and resilient under stress. Unlike transient moods, emotional stability is a stable personality trait that dictates how individuals perceive and respond to external setbacks. In the context of human–AI interaction, individuals with high stability tend to view system errors as manageable technical issues, whereas those with low stability may perceive the same errors as a threat to their situational control, triggering more volatile affective shifts. Emotional stability was assessed using the Emotional Stability Scale proposed by Chaturvedi and Chander, which includes five dimensions: pessimism and optimism, anxiety and calmness, aggression and tolerance, dependence and autonomy, and indifference and empathy (Chaturvedi and Chander, 2010).

To eliminate the influence of individual differences on the results, we divided participants into different groups. We did not simply use the traditional sum of questionnaire scores or pre-set criteria to group them. Instead, we chose a data-driven, exploratory approach. Specifically, we used an unsupervised learning group classification method proposed by Luo et al. (2025). Its core advantage lies in its ability to group and extract features based on the intrinsic structure of the data, rather than being constrained by *a priori* theoretical assumptions. This process of this method involves first projecting the high-dimensional scale data into a low-dimensional subspace using singular value decomposition (SVD) to capture the maximum variance in the data, thereby summarizing most of the information in the original dimensions using a small number of principal components. This classification is widely accepted in psychology and personality research (Buss and Finn, 1987; Eysenck, 1964). Compared to simply averaging the original dimensions, this combined approach automatically determines the contribution weight of each dimension based on the data variance, avoiding the controversial nature of subjective settings. It also makes the classification results more robust by reducing the interference of noise and redundant dimensions, and enhances the interpretability of the classification results by generating interpretable judgment intervals based on variance contributions (Tripathy et al., 2022; Wang et al., 2023). Finally, the selection of *K* = 2 was determined by evaluating the cluster stability and the elbow method following SVD dimensionality reduction, which revealed a distinct structure within the data.

### 3.3 Experimental and analysis process

All participants first signed an informed consent form and completed a pre-test questionnaire. Before the experiment began, participants underwent a standardized training phase, including (1) a 30-min introduction to the system operation, explaining the functions and usage of the AI system; and (2) a 10-min sample dialogue practice to ensure that participants were familiar with the interaction process. In the experiment process, users conducted multiple rounds of dialogue with different AI systems to complete preset task scenarios. After obtaining the consent of the subjects, we recorded the following data throughout the process: a complete dialogue log including user input and AI response, and a screen recording of students working on the system. After the experiment, the research team conducted a stimulated recall interview with each participant. By watching the screen recording data, the users were asked to describe the operation process and the subjective emotional experience of each interaction node based on the question-and-answer data with GenAI and the final summary report. The maximum duration of the experiment was set at 4 h. Participants were allowed to withdraw at any time throughout the experiment.

### 3.4 Determination of AI response quality and user sentiment

**Figure 1:**
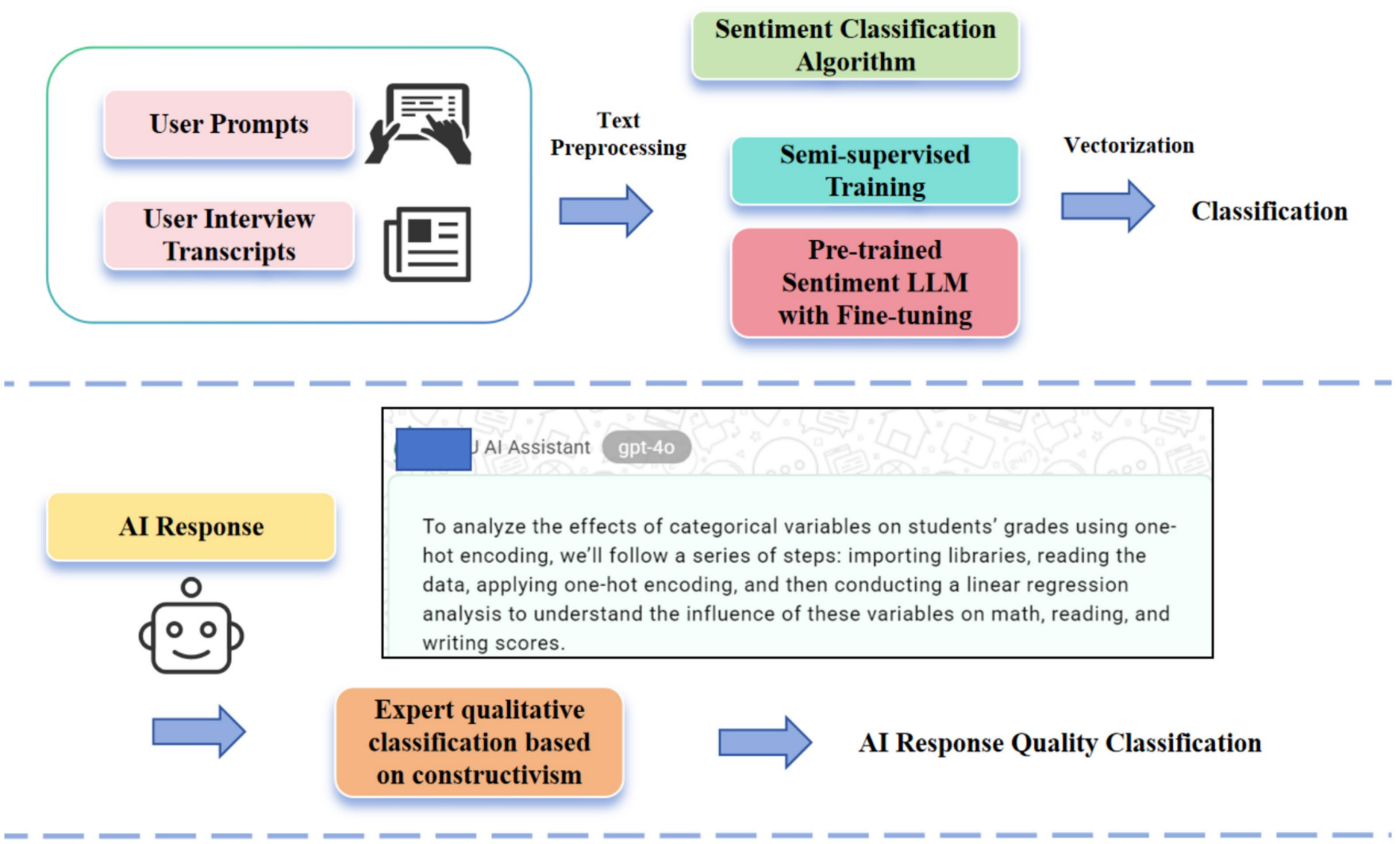
Flowchart of encoding and vectorization of data streams of different dimensions.

The core of this study is to quantify the dynamic relationship between AI response quality and user emotional state. To this end, we operationalized these two key variables. Figure 1 shows the process of the two-variable processing methods.

We define quality $Z_{t}$ as a multi-dimensional composite construct, reflecting the context-based, multi-dimensional cognitive evaluation of generative AI output by users in a specific dialogue turn. Therefore, this concept is not a single technical metric. We draw on the evaluation frameworks and design principles from the fields of human**–**computer interaction and large-scale dialogue system evaluation (Chang et al., 2024; Tam et al., 2024; Woodgate and Ajmeri, 2024), capturing the user-perceived value of the interaction through five core dimensions: relevance and helpfulness, fairness, transparency, reliability, and criticality and accountability, as shown in Table 1.

**Table 1:** A pre-structured guide to assessing AI response quality.

Dimension	Description	Key evaluation criteria
1. Relevance and Helpfulness	Evaluates whether the response directly addresses the user’s query intent and provides practically useful content.	Relevance to the user’s current query and context. The information or solutions provided are inspiring. Visible progress in advancing tasks or solving problems.
2. Fairness and Inclusiveness	Evaluates whether the response avoids discriminatory, biased, or exclusionary content.	Absence of stereotypes or biases related to gender, ethnicity, culture, etc. Use of neutral and inclusive language.
3. Reliability and Safety	Evaluates the factual accuracy and logical consistency of a response in conjunction with its potential for harm.	Factual correctness and verifiability of information. Logical coherence and internal consistency. Avoidance of generating harmful, dangerous, or unethical content.
4. Transparency and Explainability	Evaluates the degree to which the AI discloses its nature and the reasoning behind its responses.	Clarity about its identity as an AI system Ability to provide reasoning, sources, or justification for its outputs. Avoiding presentation as an incontrovertible black-box authority.
5. Criticality and Accountability	Evaluates the response’s demonstration of critical thinking and responsible ownership of its limitations.	Ability to identify potential flaws, assumptions, or limitations in the user’s query or its own knowledge. Acknowledgement of uncertainty, confidence levels, and knowledge boundaries. Providing mechanisms for correction and guiding users to further help if needed.

In this study, $Z_{t}$ is defined as formative construct because any change in any single dimension (such as transparency or reliability) directly alters the overall quality state. This means that dimensions are “defining characteristics” of a concept, rather than its “representation.” Furthermore, high correlation between formative metrics is generally not required. In AI interactions, a highly “relevant” response might lead to a decline in overall quality due to insufficient “reliability” (such as hallucination issues). Each dimension captures a different aspect of quality and cannot be substituted for another, which aligns with the characteristics of formative measurements. Moreover, logically, it is the scores of these evaluation dimensions that “combine” to form the current quality level, rather than a potential, invisible “quality trait” dominating the performance of these dimensions. Therefore, adopting formative logic is more consistent with the actual context of evaluating large-scale dialogue systems.

We selected Expert Annotation as the core evaluation method for AI response quality, stemming from the human**–**centered and interpretivist design and evaluation philosophy: three research assistants unfamiliar with the experimental hypotheses independently segmented and preliminarily cleaned each AI response in the dialogue logs. Subsequently, a panel of two human**–**computer interaction experts and one linguist independently categorized each AI response.

From an interpretivist perspective, the understanding of “quality” is socially constructed, emerging from dialogue and consensus-building among evaluators (McKay et al., 2024). Therefore, prior to independent scoring, we facilitated a guided discussion session. We did not provide the expert panel with predefined abstract definitions of “high quality” or “low quality.” Instead, we encourage experts to elaborate, based on concrete response examples, what they personally considered high- or low-quality replies and to share the reasoning behind their judgments. Notably, the experts’ perspectives converged through discussion, reflecting a shared, intuitive core definition of “quality.” The initial calibration reliability (Cohen’s *k* = 0.72) confirmed the validity of this consensus.

During the independent scoring phase, the experts comprehensively judged each AI response based on the five dimensions we identified, directly categorizing it as high, medium, or low. When the three experts disagreed with the classification, they reconsidered the response until reaching a final consensus. This holistic scoring method based on expert consensus avoids disputes over the subjective weighting of each dimension and ensures the robustness and interpretability of the classification results.

To more objectively capture the user’s immediate emotional state, we employed a sentiment computation method based on natural language processing. We used a combination of two types of sentiment input: text prompts entered by the user during the conversation and sentiment narratives from retrospective interviews. By overlaying two types of text, we can capture the user’s full emotional landscape more comprehensively and deeply. This measurement not only enhances the reliability of sentiment analysis but also enables us to more accurately align emotional changes with specific AI interaction moments.

We used the cardiffnlp/twitter-roberta-base-sentiment model (Barbieri et al., 2020) developed by the Cardiff University NLP team. This model is a variant of RoBERTa fine-tuned on a large amount of Twitter data. It directly maps input text into three discrete sentiment dimensions: negative, neutral, and positive. Although the model has been fine-tuned on a large amount of social media data, we further fine-tuned it given the unique language style and emotional expression of our conversations and interviews.

The baseline model initially achieved an accuracy of 84.2% on our specific educational inquiry corpus. After fine-tuning with the manually annotated gold-standard dataset, the model’s performance saw a 14.5% relative improvement, reaching a final accuracy of 96.4%. To provide a comprehensive evaluation as suggested, we further report the macro-averaged Precision of 0.95, Recall of 0.96, and an F1-score of 0.96. This high level of precision, particularly in distinguishing ‘Neutral’ from ‘Positive’ academic reflections, ensures the robustness of the emotional valence data used in our Markov chain analysis.”

**Figure 2:**
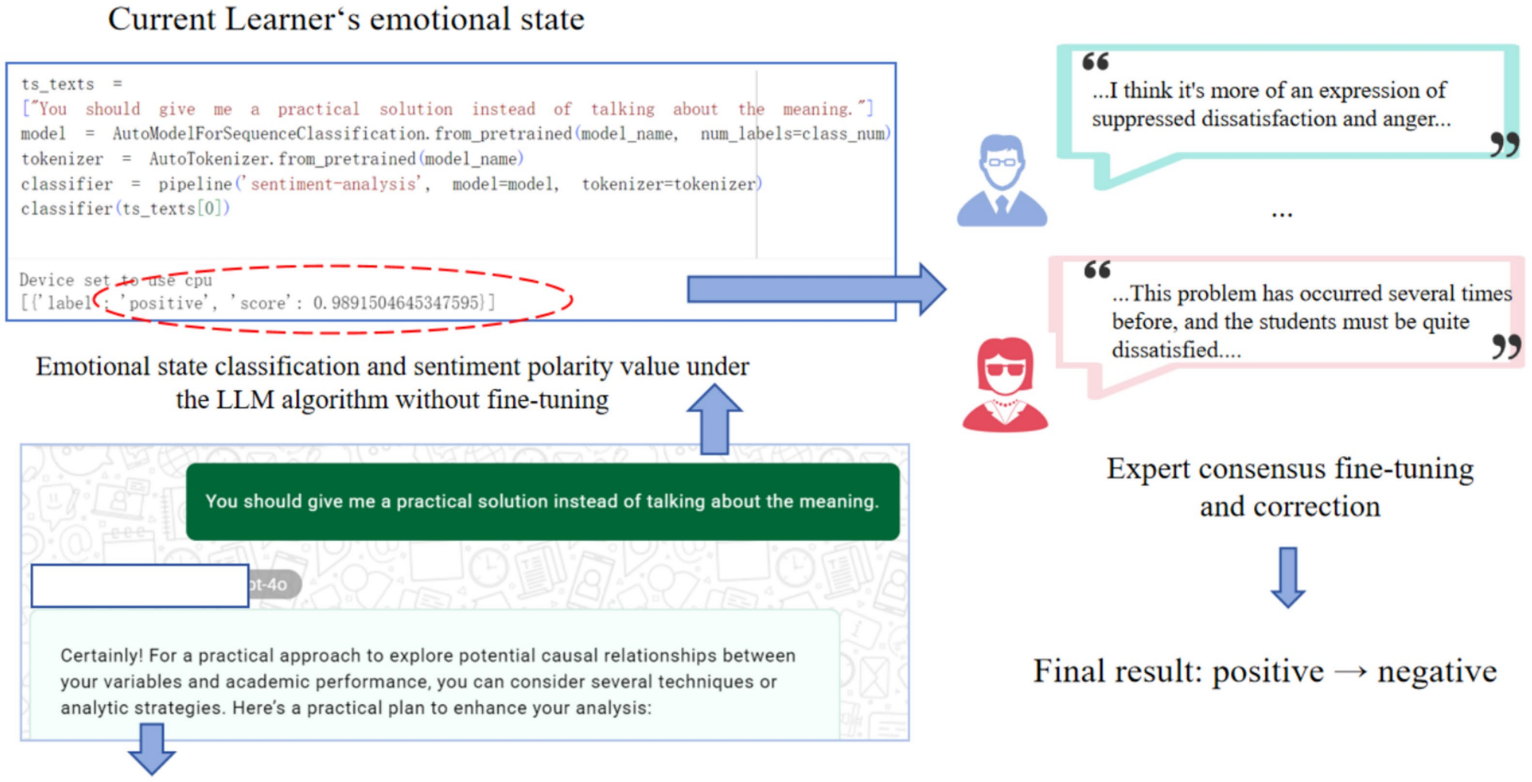
Example of sentiment score mapping and fine-tuning for large models.

In tests on the 10% validation set, accuracy improved by 14.5%. After discussion, two doctoral students in educational technology manually annotated 50 sentiment texts and used them as a training set to fine-tune the model to better adapt it to the expression style of our research corpus. Examples of these mappings are shown in Table 2, and Figure 2 shows an example discussion of fine-tuning the sentiment score.

**Table 2:** Classification examples of the pre-trained sentiment model.

Classification	Description	Example
Negative	Includes emotions such as anger, frustration, disappointment, and dissatisfaction	“I think there’s something wrong with the AI calculations that’s really annoying me.”
		“Do not use machine learning algorithms on me. The purpose of my research is not to predict.”
Positive	Includes emotions such as happiness, satisfaction, excitement, gratitude, etc.	“This is a good idea, keep going.”
		“Can you explain the solution you gave in another way?”
Neutral	Includes emotions such as calmness, objectivity, inquiry, and confirmation	“Then use cluster analysis as you said.”
		“Adjust the format of my document according to the suggestions you gave me before.”

In our model, the Neutral state is operationalized as a non-polarized state of cognitive processing. This operationalization is grounded in D’Mello and Graesser’s (2012) theoretical model of affect during learning, which identifies a functionally distinct state of cognitive engagement that is neither affectively positive nor negative, but represents focused, on-task information processing. Empirically, learners in this state tend to exhibit sustained task behavior, balanced prompt formulation, and absence of affective markers in their language, consistent with the textual patterns observed in our Neutral-labeled turns. While we acknowledge that this category may encompass a variety of subtle psychological nuances, its primary function in this research is to represent cognitive engagement, which is a steady state where the user is focused on the logic and task requirements of academic writing without a significant shift in affective valence (Huang et al., 2024). By treating neutrality as this baseline, we can more effectively isolate the transitions associated with GenAI response quality. This allows the model to identify the turns at which task-focused engagement shifts into a polarized affective valence state (either positive or negative), thereby quantifying the statistical relationship between AI output and affective valence transitions.

Regarding the operationalization of AI response quality intoordinal variables (high, medium, low), this study aims to balance statistical simplicity with the preservation of psychological meaning. Its main theoretical basis stems from the requirements of the CDMC framework. To further validate the construct validity of the three-level ordinal classification, we conducted a one-way ANOVA examining differences across the five evaluation dimensions. Results confirmed that the three quality levels (Low, Medium, High) differed significantly on all five dimensions (all *F* > 937, all *p* < 0.001), with large effect sizes. Post-hoc Tukey HSD comparisons confirmed the ordinal structure: Low < Medium < High on all dimensions (all *p* < 0.001). The composite scores were 1.85 (SD = 0.54), 3.08 (SD = 0.61), and 4.26 (SD = 0.49) for Low, Medium, and High quality respectively, providing strong empirical evidence that the three-category classification captures psychologically meaningful and statistically distinct levels of AI response quality.

While dimensions such as relevance and reliability are theoretically complex, users’ perceptions in real-time interactions are typically reflected through “overall ratings” and “threshold effects.” Integrating these aspects into ordered categories through expert consensus helps filter out fine-grained measurement noise, thereby improving the model’s ability to detect sudden emotional changes. High inter-rater reliability (Cohen’s *k* = 0.72) further validates the effectiveness of this method, highlighting the scientific rigor and reproducibility of our classification scheme, despite the need for necessary statistical simplifications.

To model how AI quality is associated with emotional state transitions, we utilize a log-linear model, incorporating covariates into the transition probability matrix via multinomial logistic regression. Using ordinal scaling ensures robustness of parameter estimation and enhances the interpretability of marginal effects at different quality levels.

### 3.5 Human–computer interaction modeling framework

According to cognitive theory, the trigger for emotional changes is the “evaluation of the current event,” rather than the accumulation of historical factors (Ellsworth, 1991; Watson and Spence, 2007). Each conversational turn can be considered an independent stimulus–response event. In our experimental context, this stimulus is the specific response generated by AI in each conversational turn. Based on this, this study adopts a human**–**computer interaction emotion transfer framework. The core of this framework is the chain of emotional states: Previous Emotional State → AI Response Quality → Next Emotional State,” which assumes a sequence relationship between a specific AI response and subsequent emotional state to quantify state changes. We adopted the principle of time alignment and regarded each change of the user’s emotion as a state transition node. We sorted the prompt words entered by all students, sorted all non-repeated, modified prompt words as emotion nodes, and extracted the retrospective descriptions of these prompt words from the retrospective interviews and incorporated them into the same emotion.

Whilea user’s overall emotional trajectory may be influenced by historical accumulation, our model focuses on quantifying the immediate, direct impact of each AI response. This simplification allows us to clearly isolate the association between AI quality and affective valence transitions, thereby more effectively answering our research questions. Furthermore, our human**–**computer interaction model separates internal states from external interventions, allowing us to treat AI response quality as an exogenous (or quasi-random) treatment effect for subsequent analysis.

**Figure 3:**
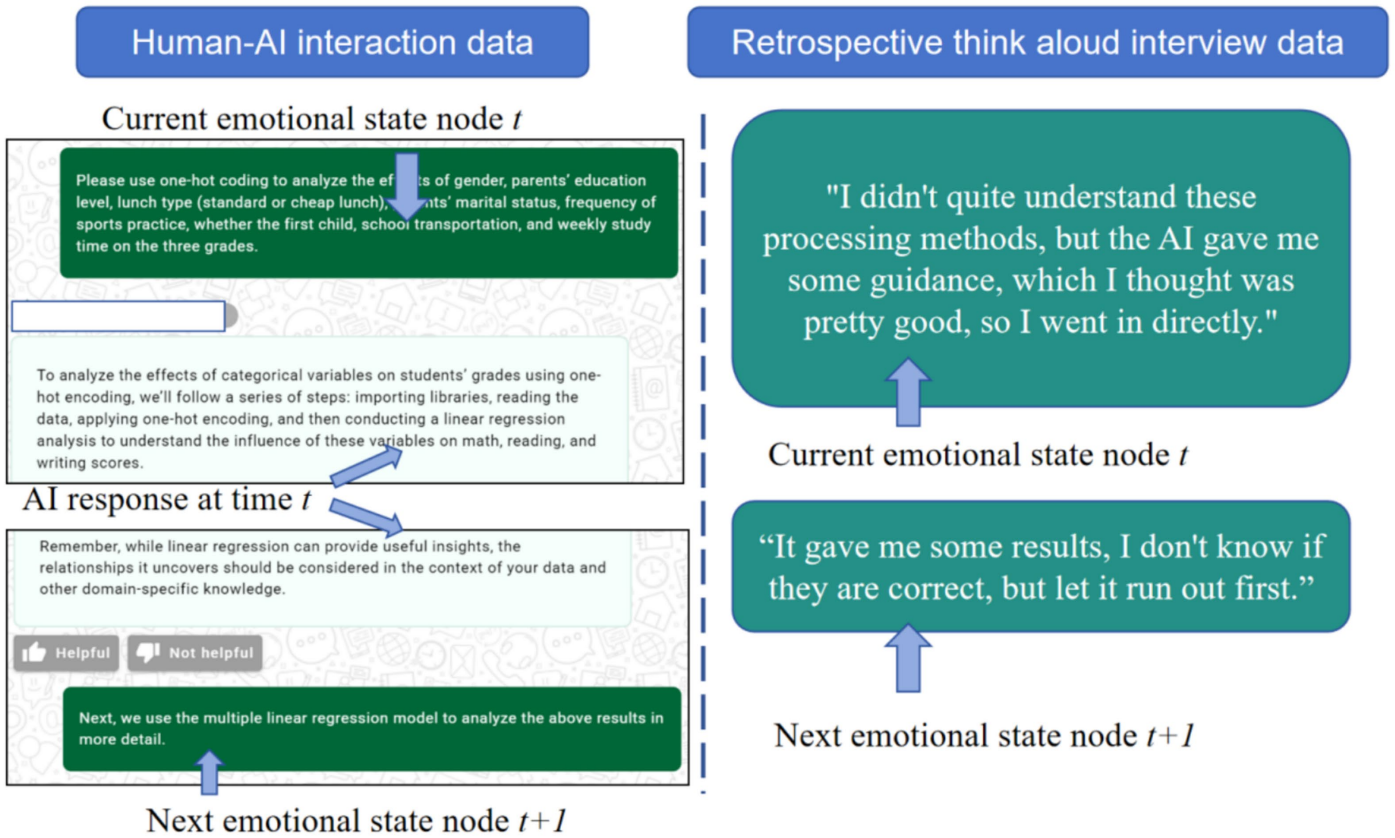
An example of time alignment of different data sources in a simulated academic writing task.

Figure 3 provides an example to illustrate this sequence dependency. At the current emotional state node *t*, the user’s interaction data inputs a specific request for using a particular analysis, while concurrent interview data shows the user’s current emotional state is one of confusion. This response influences the user’s emotional state. Finally, at the next emotional state node *t* + 1, the human**–**computer interaction data shows the next step is to execute the specific analysis, while the user’s description in the retrospective interview transforms into an initial sense of trust.

### 3.6 Covariate-dependent Markov chains

To address the dynamic complexity of human**–**computer interaction, our CDMC model explicitly incorporates three core psychological phenomena: inertia, interaction, and adaptation.

First, emotional inertia is captured through first-order Markov properties. Although the model assumes that the next state $X_{t+1}$ depends only on the current state $X_{t}$ and the covariate $Z_{t}$ , this does not mean ignoring history. Instead, $X_{t}$ acts as a psychological bond, summarizing the cumulative effect of all previous interactions. Mathematically, inertia is directly modeled by the self-transition probability, representing the baseline tendency for the emotional state to persist in the absence of sufficiently disruptive stimuli.

Second, interaction dynamics are fundamentally reflected in the conditionality of the transition matrix with respect to the covariate $Z_{t}$ . By modeling $P(X_{t+1}X_{t},Z_{t})$ , this framework describes each dialogue round as a discrete stimulus-evaluation-response cycle. The quality of AI response $Z_{t}$ , acting as a real-time situational stimulus, actively alters the probability of state transitions, reflecting how users dynamically adjust their emotional trajectories based on AI performance. Third, the adaptation problem is addressed by introducing emotional stability as a moderating covariate. This allows the model to estimate how different personality traits influence the resistance or sensitivity to state transitions. Furthermore, our reliance on the first-order assumption is theoretically based on cognitive appraisal theory (Lazarus, 1991), which posits that emotions are driven by an individual’s immediate assessment of changes in the current environment relative to their current baseline.

The original Markov model assumes: given the current state $X_{t}$ , the conditional distribution of the next state $X_{t+1}$ is independent of all prior states $X_{0:t-1}$ , as shown in Equation 1.


$$P(X_{t+1}|X_{t},X_{t-1},\ldots )=P(X_{t+1}|X_{t})$$ (1)

As shown in Equation 2, For the covariate-dependent Markov, the property is that given the current state $S_{t}$ and the current covariate $Z_{t}$ , the conditional probability of the next state $S_{t+1}$ is independent of the historical states $S_{t-1\ldots },$ and historical covariates $Z_{t-1}$ .


$$P(X_{t+1}|X_{t},Z_{t},X_{t-1},Z_{t-1},\ldots )=P(X_{t+1}|X_{t},Z_{t})$$ (2)

Equation 3 shows the probability of the system transferring to state k given the current $X_{t}$ and AI response quality $Z_{t}$ . The linear prediction is converted into a probability distribution through the softmax function of MLR.


$$P(X_{t}=j|X_{t-1}=i,Z_{t})=\frac{\exp(\alpha_{\mathrm{ij}}+\beta_{\mathrm{ij}}Z_{t})}{\sum_{k\in \{N,U,P\}}\exp(\alpha_{\mathrm{ik}}+\beta_{\mathrm{ik}}Z_{t})}$$ (3)

The proposed CDMC model explicitly addresses the interplay between internal psychological states and external technological stimuli. k∈{N,U,P} represents the possible emotional state at the next moment (negative, neutral, positive). The intercept $\alpha_{\mathrm{ij}}$ encapsulates the user’s intrinsic emotional baseline and inertia, representing the inherent transition tendency independent of AI performance. Conversely, the slope $\beta_{\mathrm{ij}}$ quantifies the marginal impact of AI response quality $\;Z_{t}$ as a situational moderator, which is the impact of a one-unit increase in the quality of the AI response $\;Z_{t}\;$ on the log-odds of transitioning from state i to state j .

By modeling the transition as a function of both the preceding state $X_{t-1}\;$ and the current stimulus $\;Z_{t}$ , the framework views emotional dynamics not as a product of AI alone, but as a continuous calibration process between the individual and the interactive environment.

To empirically evaluate the plausibility of the first-order assumption within the CDMC framework, we compared the first-order specification against a second-order extension that additionally conditions on the penultimate state. The first-order CDMC yielded AIC = 1,418.16 and BIC = 1,504.20 (18 free parameters; log-L = −691.08; *N* = 880 transitions), whereas the second-order CDMC yielded AIC = 1,433.18 and BIC = 1,685.33 (54 free parameters; log-L = −662.59; *N* = 788 consecutive triples). Both AIC and BIC favored the first-order model. In particular, the BIC difference (+181.1) constitutes a evidence by conventional thresholds (>10) that the 36 additional parameters of the second-order model do not provide sufficient predictive gain to justify their inclusion given the current sample size.

## 4 Results

A total of 46 undergraduates in their first to third year of study were recruited from two universities in Asia. Students collectively engaged in 886 sets of interactive sequence data, which generated 761 min of screen recordings and 493 min of audio interview data. The sample was composed of 26 males (56.5%) and 20 females (43.5%). In terms of disciplinary background, nearly half of the participants were from science and engineering (45.7%, 21 students), with the remainder distributed across business administration (30.4%, 14 students) and the humanities and social sciences (23.9%, 11 students).

**Figure 4:**
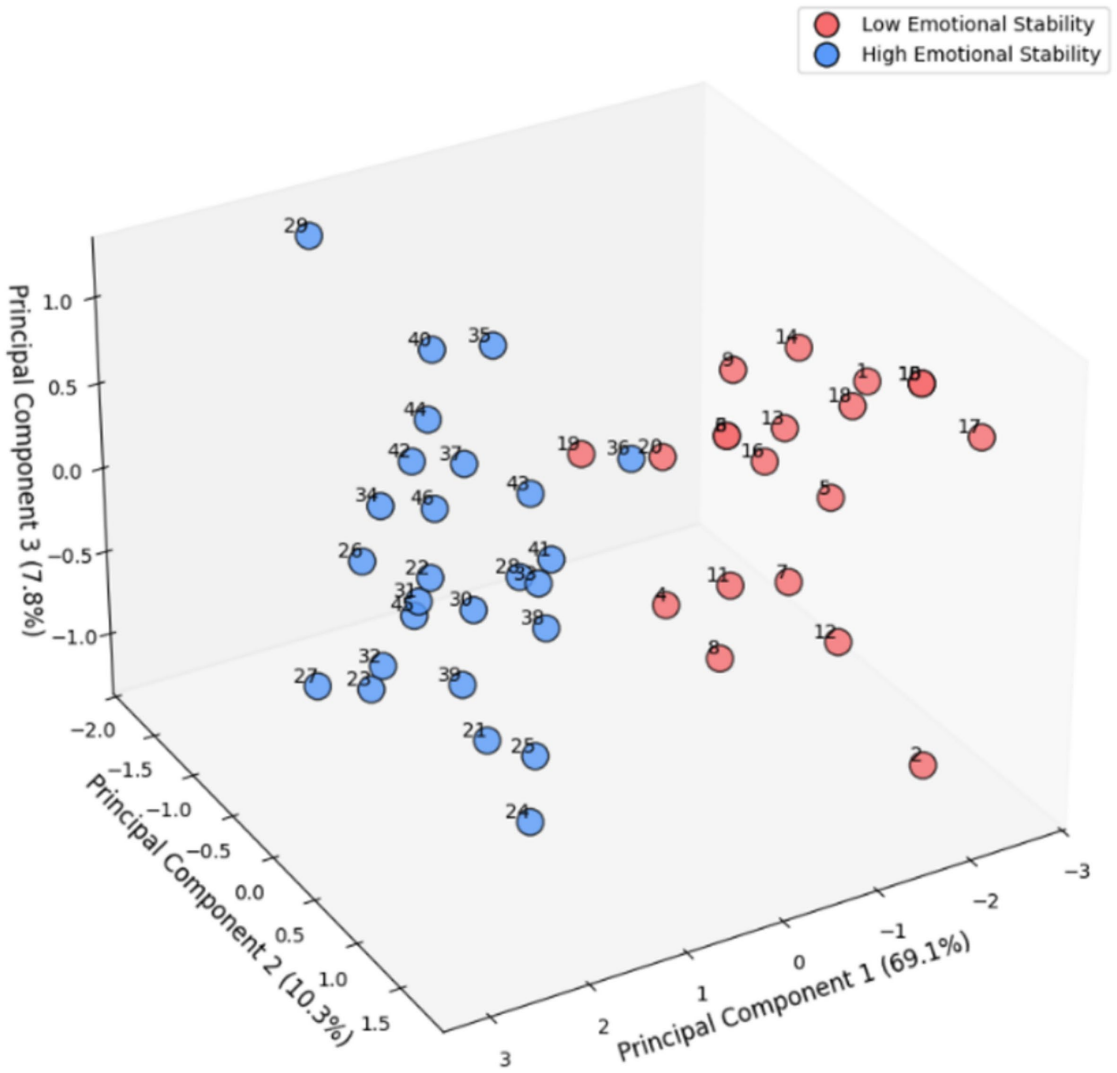
Three-dimensional graph of groups with different emotional stability based on the SVD-K-means algorithm.

As shown in Figure 4, the scores of the two clusters based on emotional stability (PC1: 69.1%, PC2: 10.3%, PC3: 7.8%) also showed significant differences (Mann–Whitney *U* = 167.2, *p* = 0.000). To confirm the psychological interpretability of the two clusters, we conducted post-hoc analyses comparing their scores across the dimensions of the Emotional Stability Scale. To validate the psychometric relevance of the two clusters, we anchored our results against the normative benchmarks established by Chaturvedi and Chander (2010). The normative study identifies a score of 181 as the minimum threshold for ‘normal’ emotional stability within the university student population (M = 187.08, SD = 12.79). In our study, the low stability cluster identified by the data-driven algorithm yielded a mean score of 171.4, which is substantively below the normative threshold.

Independent *t*-tests revealed that the groups differed significantly (*p* < 0.001) across all dimensions, including Anxiety vs. Calmness and Aggression vs. Tolerance, with large effect sizes. These results (see Table 3) demonstrate that the SVD-Kmeans procedure successfully captured qualitative shifts in affective regulation.

**Table 3:** Statistical results of pretesting emotional stability.

Dimension	Low stability cluster	High stability cluster	Cohen’s *d*	*p*-value
Total score	171.4 ± 9.2	192.7 ± 8.4	2.42	<0.001
Pessimism vs. optimism	33.5 ± 3.5	40.8 ± 2.9	2.27	<0.001
Anxiety vs. calmness	32.1 ± 3.2	41.5 ± 2.8	3.12	<0.001
Aggression vs. tolerance	31.5 ± 4.1	40.2 ± 3.5	2.28	<0.001
Dependence vs. autonomy	34.2 ± 3.8	39.5 ± 3.2	1.51	<0.001
Indifference vs. empathy	40.1 ± 4.2	30.7 ± 3.1	2.54	<0.001

**Figure 5:**
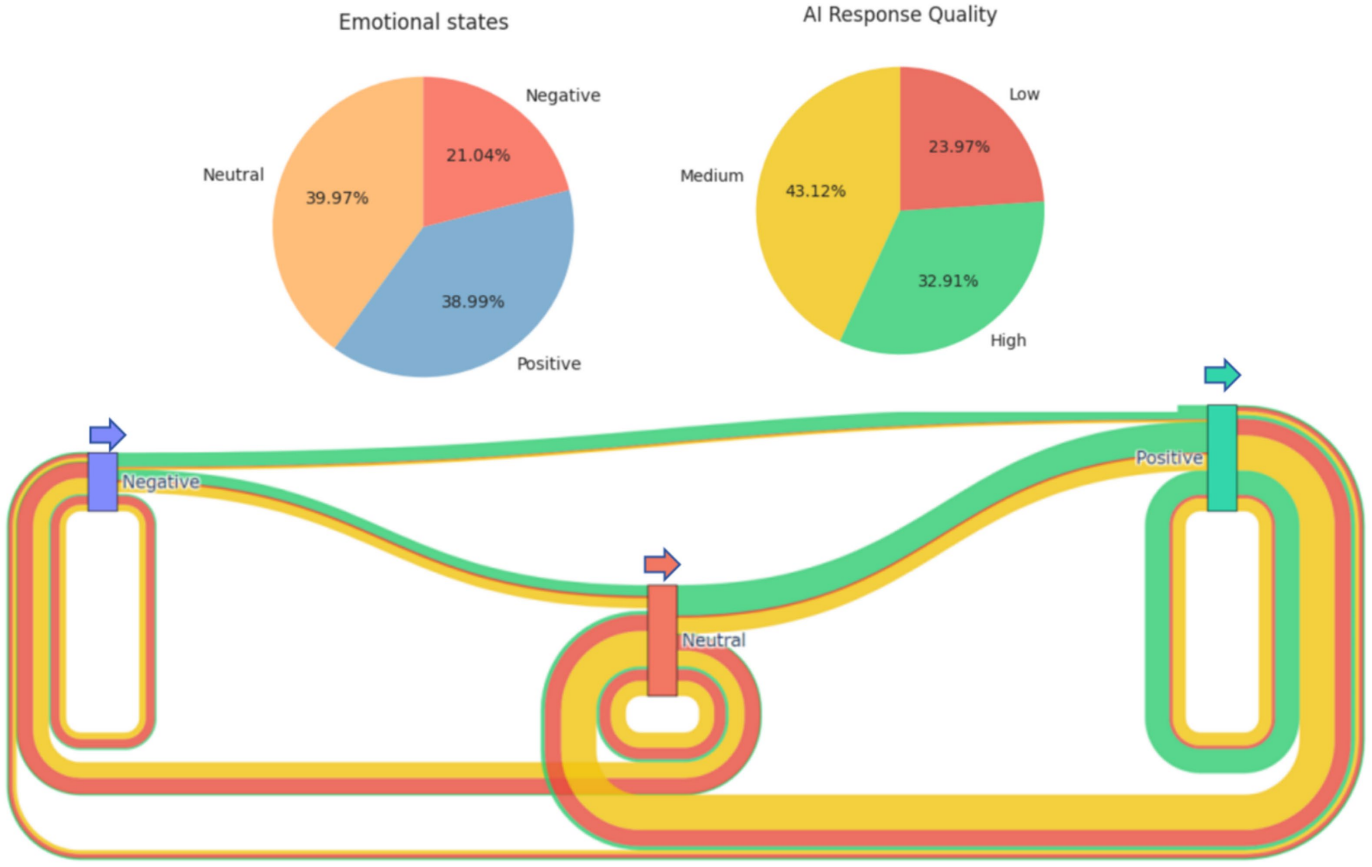
A combined chart including the proportion of emotional states and AI response quality categories, as well as the changes and flows of different emotional states.

Figure 5 shows the proportions of different states and AI response in all-time series data, and also shows a Kissan plot that includes the overall changes. The rectangular nodes in the diagram represent the three emotional states of “positive,” “neutral,” and “negative,” while the lines connecting them represent emotional transitions. Arrows in the figure point to the direction of state transition. The thickness of the line directly reflects the frequency of the emotional transition, while its color reveals the quality of the AI response that facilitated the transition (green for high quality, yellow for medium quality, and red for low quality). For example, if a yellow line from “neutral” to “negative” is thicker, it means that with medium-quality AI responses, it is more common for user emotional valence to change from neutral to negative. Conversely, if a green line from “negative” to “positive” is thicker, it indicates that high-quality AI responses are very effective in changing negative emotional valence.

### 4.1 Emotional state transfer matrix results of different user groups

As hypothesized in our proposed chain framework, the quality of AI responses is strongly associated with the emotional valence transfer patterns of different emotion stability groups. For groups with low and high emotional stability, AI response quality has a substantial association with emotional transition patterns.

In the low emotional stability group, suboptimal AI responses are statistically associated with a higher probability of valence deterioration. When exposed to low-quality AI responses, users exhibit a strong tendency to either transition to or remain in a negative valence state.

Specifically, the probability of shifting from the non-polarized neutral baseline to a negative state is 88.46%, while the transition to positive valence is statistically rare (1.92%). Medium-quality AI responses appear to partially mitigate this trend; although the transition from neutral to negative remains high (72.22%), there is a measurable increase in transitions toward neutral or positive states, suggesting a slight stabilization of affective valence. In contrast, high-quality AI responses are associated with a positive valence shift. In these instances, the probability of moving from a neutral baseline to a positive state reaches 88.0%, while the likelihood of persisting in a negative valence state is minimized to 7.02%.

**Figure 6:**
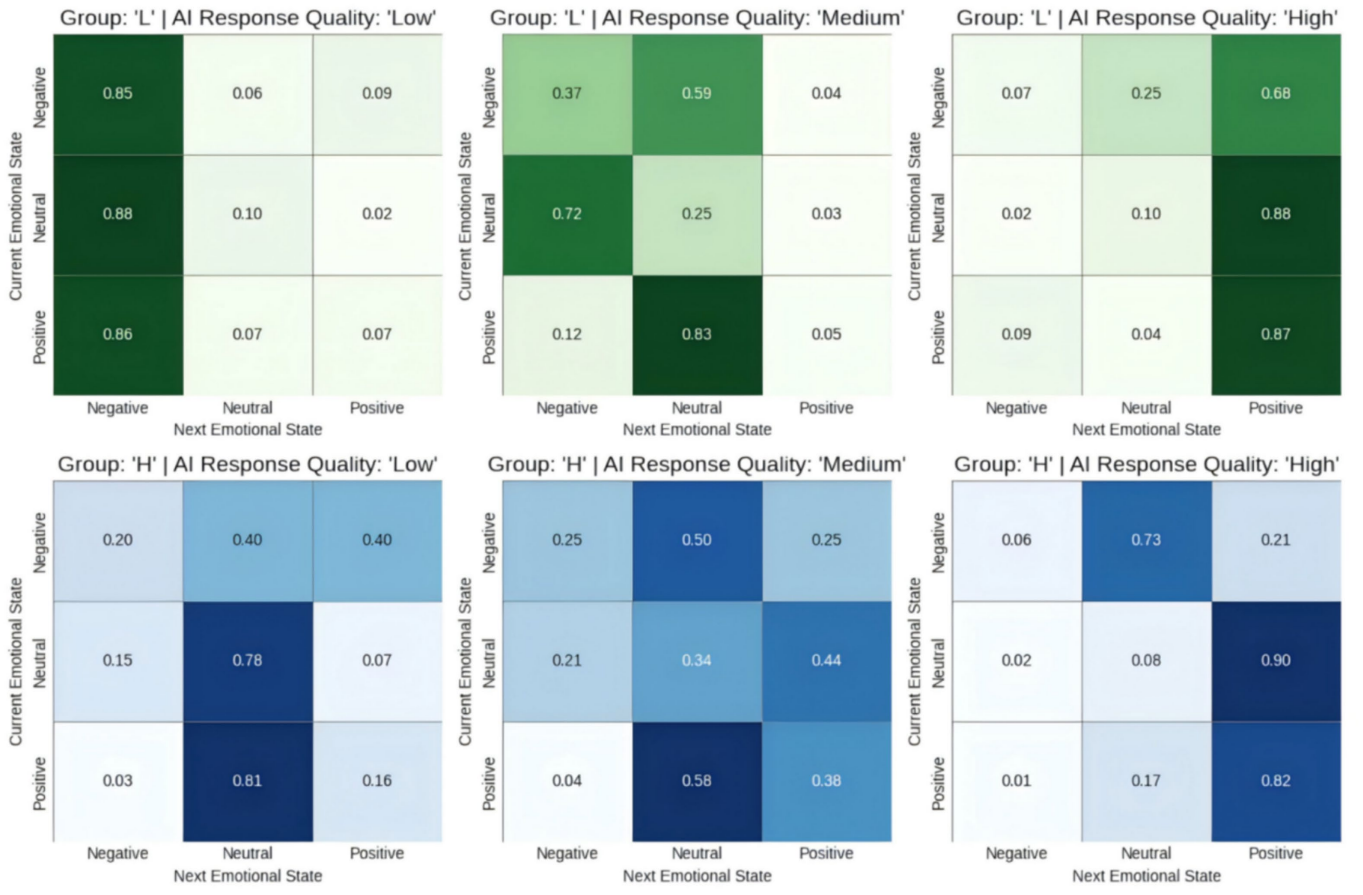
Transfer matrix diagram of different emotional stability groups (low emotional stability group L vs. emotional stability group H).

In contrast, the high-emotional stability group showed greater emotional resilience to the impact of AI quality, characterized by the maintenance of the non-polarized baseline. Figure 6 shows the emotional transition matrix for different emotional stability groups in response to AI response quality $Z_{t}$ . Low-quality AI responses do not trigger the same magnitude of negative valence shifts as seen in the low-stability group. Instead, these users show a strong tendency to remain in the neutral state (78.05%), indicating a capacity to sustain cognitive engagement despite suboptimal system performance. With medium-quality responses, their emotional patterns became more balanced. While the probability of transitioning from “neutral” to “negative” increased (21.37%), the probability of transitioning to “positive” was also quite high (44.44%). This suggests a more complex pattern of emotional transitions. Similar to the low-emotional stability group, with high-quality AI responses, this group also shows a high probability of transitioning to positive valence (90.48%) and a high maintenance rate of positivity (exceeding 80%). This suggests that while high-stability users are less prone to negative shifts, high-quality AI is still a significant factor in promoting a transition toward positive affective valence.

### 4.2 Inference analysis results under multivariate logistic regression

Before conducting inference analysis on emotional state transitions, we conducted two key validation steps to ensure the rigor of our model selection. First, we used a chi-square test of independence to verify whether AI Response quality significantly influences emotional transitions. Preliminary results showed a significant correlation between AI response quality and the user’s next emotional state (*p* < 0.001, chi-square statistic: 435.26, df: 16). This provides statistical support for AI response quality as a core predictor variable.

Because the initial status was missing data for the previous time point, it was deleted before performing the multivariate logistic regression. We used a multi-nominal logistic regression model to predict state transitions. Tables 4, 5 show the joint impact of AI quality and the user’s current emotional state on emotional transitions. The model results were grouped and analyzed based on student academic performance. The baseline reference category for emotional state was set to “neutral,” and the reference category for AI response quality was set to “high.” Beta coefficients represent the log-odds change relative to the baseline reference category.

**Table 4:** Results of multi-distance logical retrospective analysis of the low emotional stability group.

$X_{t+1}$ = negative	*β*	SD	*z*	*p* > |*z*|	CI lower	CI upper	Sig
$X_{t}$ = Neutral	0.7572	0.389	1.945	0.052	−0.006	1.52	†
$X_{t}$ = Positive	−0.9346	0.373	−2.507	0.012	−1.665	−0.204	*
$Z_{t}$ = Low	3.5019	0.604	5.802	0	2.319	4.685	***
$Z_{t}$ = Medium	0.6522	0.49	1.331	0.183	−0.308	1.612	
Constant	−1.186	0.47	−2.525	0.012	−2.107	−0.265	*

$X_{t+1}$ = positive	*β*	SD	*z*	*p* > |*z*|	CI lower	CI upper	Sig
$X_{t}$ = Neutral	1.0131	0.462	2.192	0.028	0.107	1.919	*
$X_{t}$ = Positive	0.44	0.49	0.898	0.369	−0.52	1.4	
$Z_{t}$ = Low	−2.0561	0.648	−3.175	0.001	−3.325	−0.787	**
$Z_{t}$ = Medium	−4.4243	0.518	−8.536	0	−5.44	−3.408	***
Constant	1.2397	0.302	4.11	0	0.648	1.831	***

**Table 5:** Results of multi-distance logical retrospective analysis of the high emotional stability group.

$X_{t+1}$ = negative	*β*	SD	*z*	*p* > |*z*|	CI lower	CI upper	Sig
$X_{t}$ = Neutral	0.4764	0.7	0.681	0.496	−0.895	1.848	
$X_{t}$ = Positive	−1.2946	0.77	−1.681	0.093	−2.804	0.215	†
$Z_{t}$ = Low	0.2321	0.732	0.317	0.751	−1.203	1.667	
$Z_{t}$ = Medium	1.1635	0.685	1.698	0.089	−0.179	2.506	†
Constant	−2.178	0.598	−3.641	0	−3.35	−1.006	***

$X_{t+1}$ = positive	*β*	SD	*z*	*p* > |*z*|	CI lower	CI upper	Sig
$X_{t}$ = Neutral	2.9083	0.48	6.058	0	1.967	3.849	***
$X_{t}$ = Positive	2.4689	0.471	5.245	0	1.546	3.392	***
$Z_{t}$ = Low	−3.6245	0.398	−9.097	0	−4.405	−2.844	***
$Z_{t}$ = Medium	−1.8193	0.29	−6.263	0	−2.389	−1.25	***
Constant	−0.8683	0.395	−2.201	0.028	−1.642	−0.095	*

For participants in the low emotional stability group, AI response quality was a significant predictor of valence shifts. Multiple regression results showed that low-quality AI responses had a significantly negative impact on emotional valence transfer compared to high-quality AI.

Low-quality AI responses, relative to high-quality ones, were associated with a significant increase in the log-odds of shifting to a negative valence (*β* = −2.0561, *p* = 0.001) and a concurrent decrease in the log odds of shifting to a positive valence (*β* = −2.0561, *p* = 0.001). These results indicate that low-quality interactions are significantly associated with downward valence transitions. Medium-quality AI responses did not significantly predict a shift to negative valence (*β* = 0.6522, *p* = 0.183) but showed a substantial negative association with the transition to positive valence (*β* = −4.4243, *p* < 0.001), suggesting that mediocre AI performance primarily acts by inhibiting positive valence recovery.

In contrast to the low emotional stability group, participants in the high emotional stability group demonstrated greater emotional resilience. Results showed neither low-quality nor medium-quality AI responses significantly predicted a transition to negative valence (Low-quality AI Response: *p* = 0.751; Medium-quality AI Response: *p* = 0.089).

This contrasts sharply with the results for the low emotional stability group, suggesting that this group is better able to cope with and tolerate suboptimal interactions. Neither low-quality nor medium-quality AI responses significantly predicted a transition to negative valence (Low: *p* = 0.751; Medium: *p* = 0.089). This statistically confirms that high-stability individuals are less likely to deviate from a non-polarized state when facing suboptimal AI. However, AI quality remained a critical predictor for upward valence shifts. Both low-quality (*β* = −3.6245, *p* < 0.001) and medium-quality (*β* = −1.8193, *p* < 0.001) AI responses were significantly associated with a reduced likelihood of transitioning to positive valence, reinforcing that high-quality AI is necessary to facilitate positive affective shifts even in resilient users.

## 5 Discussion

This study investigates the dynamic relationship between AI response quality and emotional state transitions within GenAI-assisted learning contexts. Utilizing a chain interaction framework and real-time interaction data, we move beyond static self-report measures to quantify the statistical association between AI response characteristics and the emotional valence trajectory of users. In this section, we focus on the valence shifting patterns of AI quality (Section 5.1) and the resilience and susceptibility linked to emotional stability (Section 5.2), which together explain the evolution of user affective valence during GenAI interaction. In Section 5.3, we provide insights into the application of human**–**computer interaction in education. It should be noted that the transition probabilities reported here were derived partly from stimulated recall data, which, while anchored to screen recordings, may be subject to outcome-congruent reappraisal. Participants who ultimately succeeded in their writing tasks may have retrospectively evaluated earlier neutral or ambiguous turns more positively than they were experienced in the moment. This suggests that the reported probabilities of positive valence transitions, particularly those following high-quality AI responses, should be interpreted with some caution, as they may represent a modest upward bias relative to in-the-moment affective experience.

### 5.1 Emotional transfer patterns are highly dependent on the quality of AI responses

Our analysis of the interaction sequences reveals that AI responses function as a critical information stimulus that is strongly associated with the direction of subsequent valence shifts. The transformation matrix shows that all interaction sequences exhibit a clear polarization trend. This also means that the quality of the AI response is strongly associated with whether the neutral baseline state is maintained or shifts toward a polarized valence. When AI response quality is high, transitions are primarily characterized by an upward shift toward positive valence, manifesting as a high probability of transitioning from negative to positive and the robust maintenance of the positive emotional state. This suggests that high-quality AI responses may serve as affective stabilizers. Conversely, low-quality AI responses are associated with downward valence shifts, with users more frequently transitioning from a non-polarized neutral baseline to a negative state.

This polarized pattern is best explained by the Control-Value Theory (Pekrun, 2006). Users continuously appraise the quality of information received: when AI output is perceived as ineffective or erroneous, it may be interpreted as a loss of situational control, leading to a shift toward negative valence (e.g., frustration or anxiety). In contrast, high-quality AI responses provide valuable, accurate information that reinforces their sense of competence and control over the information acquisition process, which is associated with more positive affective valence (Kohnke and Moorhouse, 2025).

Notably, our results show that for high-stability users, suboptimal AI responses still significantly hinder the transition to positive valence (*β* = −1.8193, *p* < 0.001). This clearly suggests that to achieve a shift to emotions such as fun and satisfaction, which have been shown to drive user satisfaction, even users with high emotional stability still need high-quality, high-expectation AI responses to provide a new sense of control and value.

### 5.2 Emotional stability as a modulator of information tolerance

This study identifies emotional stability as a key factor shaping the relationship between information stimuli (AI quality) and valence transitions. By isolating this trait within dynamic interaction sequences, we provide empirical evidence of how individual differences influence the tolerance of suboptimal information.

Based on norm comparison, the low-stability group in this study belongs to the vulnerable population in a psychological sense. For the low emotional stability group, low-quality AI responses are linked to a high probability of valence deterioration. Regression analysis (Table 4) indicates that low-quality AI significantly increases the likelihood of these users shifting to negative valence. In stark contrast, high-stability users demonstrate higher affective resilience. As shown in Table 5, neither low- nor medium-quality AI responses were significant predictors of a downward shift toward negative valence.

For participants with high emotional stability, the neutral state is stable. When faced with low-quality AI, these users demonstrate a 78.05% probability of remaining in the neutral baseline instead of shifting to negative valence. This suggests that high-stability individuals possess a higher threshold. High emotional stability users tend to employ proactive emotion regulation strategies such as cognitive reappraisal (Brockman et al., 2017). They can re-evaluate the AI system’s errors or so–so responses, interpreting them as tolerable external system limitations rather than evidence of failures in their own behavior or abilities. This allows them to cope better with and tolerate suboptimal interactions. This cognitive strategy effectively prevents the generation of negative emotional valence, thereby maintaining a relatively positive emotional state (Wolgast et al., 2011), enabling them to better cope with and tolerate suboptimal interaction experiences.

In contrast, for the low emotional stability group, the neutral baseline is highly volatile. The lack of a buffer means that even medium-quality AI responses significantly stifle the transition from neutral to positive valence, essentially trapping these users in a non-positive cycle. This highlights that while high-stability users use the neutral state as a buffer, low-stability users experience it as a fragile transition point that easily succumbs to valence deterioration.

### 5.3 Implications for educators and system designers

Given these characteristics, we recommend that curriculum designers and AI developers should adopt emotional support and maintenance of a sense of control as core design goals for GenAI-assisted learning environments. To maintain learners’ sense of control and value, the AI system should not only provide answers but also offer brief explanations or operational paths to let learners understand how the AI arrived at the answer. This transparency helps learners revise their mental models and reduces the loss of situational control caused by unpredictable AI behavior. Furthermore, given that users with different emotional stability may have different needs for GenAI-supported learning processes, the system should also integrate real-time emotion recognition tools and provide personalized interventions for users with different emotional stability. For example, for those with low emotional stability, more simplified questions, step-by-step prompts, or answers reframed in a more supportive tone can be adopted. This immediate feedback mechanism helps restore learners’ situational control during the initial stages of emotional deterioration, thereby improving their learning effectiveness. For learners with high emotional stability, the system can focus on building processes that enhance creativity and reflection to help them develop higher-order cognitive skills such as critical thinking.

In the era of AI-assisted learning, the importance of emotional stability as a key learning ability may be underestimated. Therefore, we also recommend that educators and teachers, when applying GenAI tools, should not only focus on technical operation or cognitive content but also actively cultivate students’ emotion regulation strategies. This can be achieved by, for example, encouraging them to attribute errors to technological imperfection rather than personal inadequacy, or by openly discussing and reflecting on instances of challenging GenAI interactions in the classroom to improve students’ tolerance for variable interaction quality and maintain their learning interest.

## 6 Limitations

This study uses Covariate-Dependent Markov Chain to explore the emotional dynamics of students with different emotional stability during interactive learning with AI, providing a new perspective for studying the interactive process in GenAI-assisted learning. However, this paper still has some limitations. First, the findings of this study are limited by the characteristics of the sample and the context and may not be sufficient to generalize the GenAI interaction patterns between different disciplines.

Second, the sample is mainly composed of undergraduate students from Asian universities, which may limit the generalizability to different cultural backgrounds, educational levels, and informal learning environments. To ensure the statistical power and convergence of the Markov transition matrices, we discretized complex emotional experiences into three core affective polarities (Positive, Neutral, and Negative). This simplification may lead to fine-grained emotional nuances being merged into broader categories, potentially inflating the reported transition probabilities.

Furthermore, measuring and operationalizing emotional stability as a personality trait has certain psychometric limitations, but discretizing this continuous personality variable using K-means clustering may mask more subtle differences in trait effects. There is also a possibility of overlooking other important variables, leading to unstable results.

To ensure the statistical power and convergence of the Markov transition matrices, we discretized complex emotional experiences into three core affective polarities (Positive, Neutral, and Negative). This simplification may lead that fine-grained emotional nuances are merged into broader categories, potentially inflating the reported transition probabilities. Valence alone may not be sufficient to reflect the full picture of emotional state. Future research should explore more granular multidimensional models.

In addition, the statistical basis of this study is based on the Markov property, focusing mainly on the moment-to-moment emotional transfer pattern. This design lacking longitudinal data may underestimate the importance of cumulative effects, such as the continuous improvement of students’ experience and abilities in the long-term GenAI interaction cycle.

Last, in natural settings, a user’s prior emotional state may influence the quality of their prompts, which in turn affects the quality of the AI’s output, creating a feedback loop. While our stimulus recall procedure anchored participants’ recollections to screen recordings and interaction logs, this largely mitigated the free-recall bias prevalent in purely retrospective self-reports. However, it did not completely eliminate post-hoc re-evaluation of ambiguous emotional states. Two specific forms of bias deserve examination in conjunction with the current findings. The first is outcome consistency re-evaluation. Participants who successfully completed the academic writing task may systematically reinterpret previously neutral or ambiguous conversations as more positive, while those who encountered persistent difficulty may do the opposite. In our transition matrix, this would manifest as a directed inflation of the probability of positive emotions in subsequent interactions, particularly after high-quality AI responses.

The second issue is cue quality confounding. In natural interaction environments, a user’s prior emotional valence state may influence the phrasing quality of subsequent cues, thereby affecting the quality of the AI output. This two-way feedback loop means that the quality of the AIresponse cannot be considered entirely independent of the user’s emotional trajectory, and our modeled time series may underestimate the extent to which users co-construct the quality of interactions through their own emotional states. Future research should employ more controlled experimental designs, such as randomly assigning pre-defined AI response quality conditions, in order to more clearly separate this bidirectional path.

## 7 Conclusion

This study breaks away from the static perspective on learner affective valence in traditional research, focusing on the affective valence dynamics of GenAI-assisted learning environments. It successfully reveals the complex patterns of emotional state transitions and key influencing factors in human**–**computer interaction. By incorporating the core interaction factor of AI response quality as a covariate in a Markov chain model, we statistically modeled the associations between AI response quality and the probability of emotional valence transitions and distinguished differences in sensitivity between learners with different levels of emotional stability. This methodological innovation provides a new analytical paradigm for future research on dynamic human–computer interaction. Our findings demonstrate that the quality of AI responses is a significant predictor of user affective valence. Specifically, AI response quality is linked to polarized valence transitions: high-quality responses are associated with an upward shift toward positive affective states, while low-quality responses frequently correlate with a downward shift toward negative valence. We urge that the design of relevant AI systems incorporate emotional support as a core principle. Furthermore, educators should actively cultivate students’ emotional regulation strategies to ensure that learners with diverse performance and personal characteristics have a high-quality AI-assisted learning experience. Looking ahead to future research, it is necessary to further deconstruct and quantify the underlying components of AI response quality.

## Data Availability

The raw data supporting the conclusions of this article will be made available by the authors, without undue reservation.

## Appendix A

Students were assigned a data science exploratory task. The researchers provided students with a public dataset related to educational academic performance from Kaggle (The data set was adjusted according to the actual situation), which included some students’ grades and personal characteristics. Students were asked to explore the potential insights in this educational dataset. At the same time, the researchers informed the students that they would use a designated LLM model to assist in the exploration and asked them to organize these findings. The specific requirements were presented again in written form as follows:

*“Task: The educational system is increasingly recognizing the importance of data in improving student performance. By analyzing various student characteristics, such as demographics, study habits, educators and institutions, we can develop strategies to foster a more personalized and effective learning environment. We will provide a relevant data set containing key variables. Please complete a 1000-2000-word essay by asking questions to the LLM tool. Please complete the data of the essay according to the table content at the bottom of the document. Please pay attention to the selection of the topic of the essay, such as innovation, etc., as well as the integrity of the structure and the logical coherence of the essay. The following data can be presented in the content of the essay through tables, pictures, etc. Please pay attention to checking the logic of the content during the generation process of the LLM tool.”*
